# A Unique and Rare Presentation of Adult Congenital Heart Disease: Common Atrium Associated with Coronary Aneurysms and Fistula

**DOI:** 10.1155/2019/3961323

**Published:** 2019-04-14

**Authors:** Muhammad Shabbir Rawala, Alex Munoz, Mohammad Hassan Pervaiz

**Affiliations:** ^1^Department of Internal Medicine, WVU-Charleston Division, Charleston, WV, USA; ^2^Department of Radiology, University of Kentucky, Louisville, KY, USA; ^3^Department of Cardiology, WVU-Charleston Division, Charleston, WV, USA

## Abstract

An atrial septal defect is the second most common congenital heart disease found in adults with a female to male ratio of 4 : 1. However, it is rare to have a complete absence of the interatrial septum (IAS) to be diagnosed in an elderly patient associated with other coexisting anomalies. We present a case of a 60-year-old female presenting with common atrium, coronary arteriovenous fistula, and coronary artery aneurysms. This case highlights rare adult congenital cardiac anomalies and the importance of thorough workup to evaluate for the intracardiac shunt in a patient who has right heart enlargement and development of pulmonary disease in adulthood without a significant history of chronic smoking. A 60-year-old female patient presented with substernal chest pain. The nuclear stress test showed no reversible ischemia; however, right ventricle (RV) dilation was present. The patient underwent further evaluation for RV dilation with a transthoracic echocardiogram that demonstrated a complete absence of IAS and was confirmed by a positive bubble study. The patient had an invasive angiography that showed severely elevated RV pressure. Oxygen saturation in the right atrium was higher than in the inferior vena cava. Hence, an intracardiac shunt with a 10% increase in oxygen saturation was identified. It also identified aneurysmal coronary arteries (measuring 0.8 to 1.0 cm). Cardiac computed tomography angiogram was performed that identified all coronary arteries to be ectatic/aneurysmal measuring up to 8-10 mm, an absence of IAS, and a possible fistula between the distal left anterior descending and a coronary vein. To our knowledge, this is the first-ever presentation of a complete congenital absence of IAS in a patient who has survived into adulthood with the development of severe pulmonary hypertension without Eisenmenger syndrome. It is unclear at this point if surgical treatment to correct the anatomical defect (which would be probably palliative) would be superior to conservative medical therapy. Besides, the presence of coronary arteriovenous fistula would make the decision-making process more complex regarding surgical versus conservative management.

## 1. Introduction

Single or common atrium (CA) is a rare congenital abnormality arising due to a complete absence of the interatrial septum (IAS). It was initially thought to be an entity accompanied with endocardial cushion defect and was first described by Young and Robinson [1]. The CA can occur as part of skeletal and muscular malformation syndromes such as the Ellis-van Creveld syndrome [2]. There are few isolated cases of CA that have been reported in young adults; however, we report a case of an elderly woman with a single atrium accompanied with coronary arteriovenous fistula and coronary artery aneurysm.

## 2. Case Report

We present a case of a 60-year-old female who presented to the hospital with complaints of dull substernal chest pain. She had chronic atrial fibrillation, chronic obstructive pulmonary disease with home oxygen, osteoarthritis, and anxiety disorder as her comorbid conditions. On examination, she did have chronic dyspnea and was on home oxygen. She did not have any cyanosis, palpitations, paroxysmal nocturnal dyspnea, or orthopnea.

**Figure 1:**
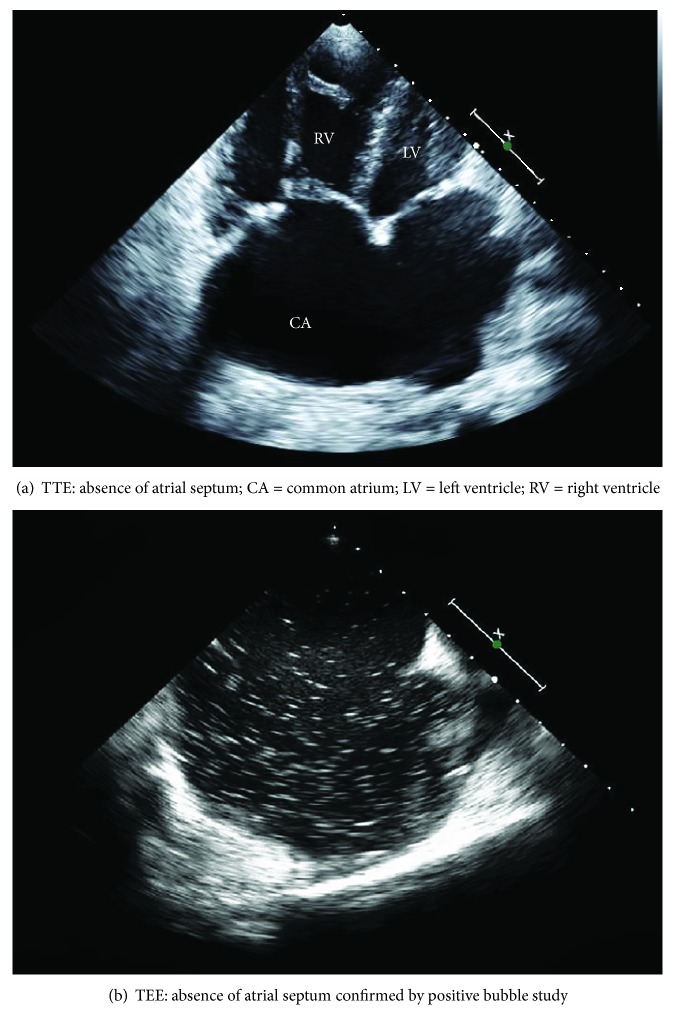
Echocardiography images revealing common atrium. (a) TTE: absence of interatrial septum. (b) TEE: positive bubble study.

She was initially evaluated with a nuclear stress test that did not show any reversible ischemia but dilation of the right ventricle (RV); ejection fraction was identified to be 54%. The patient was further evaluated by a transthoracic echocardiogram (TTE) in order to evaluate the RV dilatation. TTE identified a complete absence of IAS and a CA (Figure 1(a)). The findings were confirmed with a positive bubble study (Figure 1(b)). Transesophageal echocardiogram (TEE) was performed that confirmed the absence of IAS, demonstrated free mixing of color flow, moderate to severe tricuspid regurgitation, normal mitral valve structure, normal left ventricular ejection fraction, and enlarged right atrium (RA) and RV.

**Figure 2:**
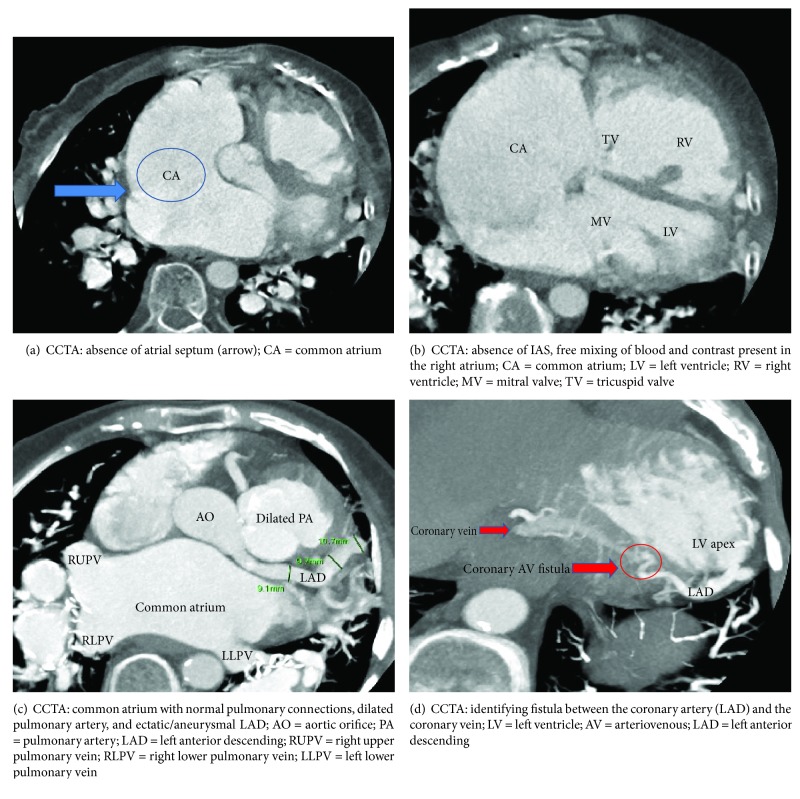
Cardiac computed tomography angiography images. (a) Absence of the atrial septum (arrow). (b) Absence of interatrial septum showing free mixing of blood and contrast present in the atrium. (c) Common atrium with normal pulmonary connections, dilated pulmonary artery, and ectatic/aneurysmal left anterior descending artery. (d) Identifying fistula between the coronary artery and the coronary vein.

The patient was evaluated with cardiac computed tomography angiogram (CCTA) that demonstrated the right coronary artery to be the dominant artery, all coronary arteries to be ectatic/aneurysmal and measuring up to 8-10 mm, a complete absence of IAS, marked dilation of CA and both ventricles, a coronary arteriovenous fistula (CAF) between the distal left anterior descending and coronary sinus, massive dilation of pulmonary arteries, and no mitral or aortic valvular abnormalities; left ventricular ejection fraction was measured to be 59% (Figures 2(a)–2(d)).

**Figure 3:**
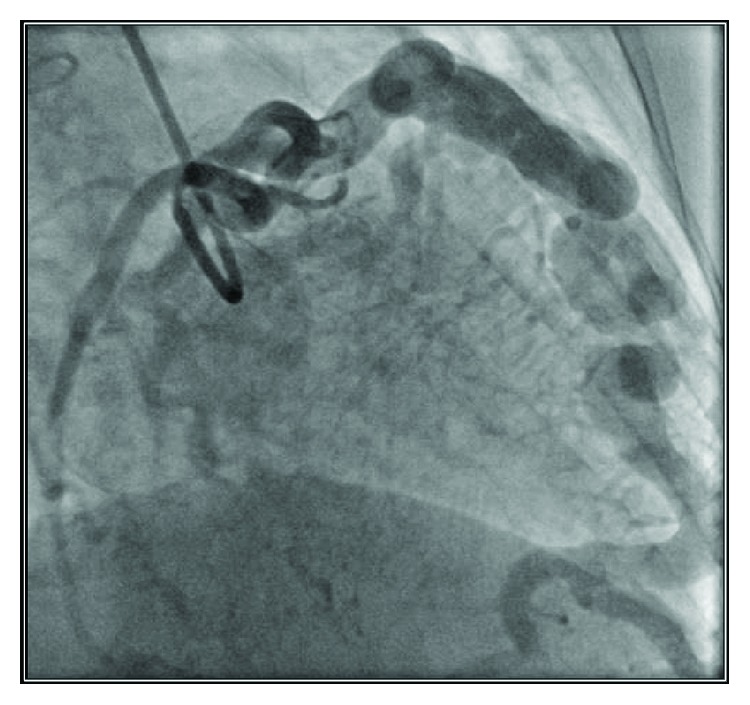
Invasive angiography revealing ectatic/aneurysmal arteries.

The patient underwent an invasive angiography (IA) which demonstrated many abnormal findings. It showed that the patient had coronary artery aneurysms measuring 0.7 cm to 1 cm (Figure 3). IA was instrumental in taking measurements regarding oxygen saturation and pressure at multiple levels identifying a large interatrial shunt with a 10% increase in oxygen saturation from IVC to RA. IA measured RV pressure to be 98/5 mmHg denoting severe pulmonary hypertension, RV end-diastolic pressure at 12 mmHg, mean RA pressure as 10 mmHg, and left ventricular (LV) end-diastolic pressure as 6 mmHg; oxygen saturation in the inferior vena cava (IVC) was 68.3%; oxygen saturation in RA was 79.8%; oxygen saturation in RV was 79.1%, and oxygen saturation in the femoral artery was 88%.

The patient had survived into adulthood with these congenital abnormalities. The patient did not have any muscular, skeletal, ophthalmologic, or vascular abnormalities to signify that her abnormalities were part of any congenital syndrome. Cardiothoracic surgery had been consulted; however, due to the technical implications of surgery, the patient was managed conservatively with no intervention to correct the congenital abnormalities. The patient was not considered for a transcatheter approach of fixing the atrial septal defect as there was a complete absence of the septum. The decision to approach conservatively also included factors such as the age of the patient, comorbid conditions, and the ability of the patient to tolerate this defect (without Eisenmenger syndrome).

## 3. Discussion

The CA is a rare entity that has been classified as being part of the endocardial cushion defect. Rastelli et al. [3] described it as (1) complete absence of the atrial septum or its representation by a small strand of tissue present in the cephalad wall of the common chamber, (2) absence of interventricular communication, and (3) an accompanying cleft in the anterior leaflet of the mitral valve. Our case did not demonstrate any abnormality of the mitral valve and was confirmed with TEE.

Levy et al. [4] reported CA as a specific and individual entity. They recommended that the term single or common atrium should be used to denote the condition characterized by (1) complete absence of the atrial septum, (2) absence of malformation of the AV valves, and (3) absence of interventricular communication.

Patients who present with CA usually report symptoms that are related to the large atrial septal defect. They can present with dyspnea on exertion, hypoxia, cyanosis, and developmental delay. Patients mostly are diagnosed during early years; however, few case reports described patients progressing to young adulthood before being diagnosed with CA [5–10]. Echocardiography is usually the first step in diagnosing CA [10]. IA and further investigative methods do help in the evaluation of coexistent congenital abnormalities. Our case had a coronary arteriovenous fistula and coronary artery aneurysms. Our case had survived to 60 years of age before this congenital abnormality was discovered to be the cause of her chronic dyspnea. The patient had not been assessed by echocardiogram or any cardiologist prior to her current presentation for evaluation of intracardiac shunt even though she had developed pulmonary disease in adulthood without a significant history of chronic smoking.

Coronary artery aneurysm (CAA) had been previously reported in 1.4% of postmortem examinations [11]. Due to routine use of IA, more cases of CAA have been diagnosed and reported. The incidence of coronary artery aneurysm has been reported to be 0.3% to 4.9% [12]. Atherosclerotic coronary artery disease accounts to be the etiology in the majority of cases [13]. Most of the patients remain asymptomatic; however, CAA does behave as arterial aneurysms elsewhere in the body and can lead to complications such as rupture, thrombus formation, and embolization. To the best of our knowledge, case reports in the literature have described the rupture of CAA if 3 cm or greater but no major case series or guidelines are established. There is also no significant consensus regarding the treatment of CAA due to the rarity of this condition and associated congenital abnormalities. Some cases in literature recommend antiplatelet along with anticoagulation to prevent thrombus formation whereas others recommend surgical resection and bypass graft surgery to prevent the risk of rupture [14].

CAF is quite rare with an incidence of 0.13% to 0.22% in patients undergoing invasive coronary angiography [15, 16]. Common etiologies of CAF are congenital, trauma, cardiac surgery, and angioplasty. It is thought that fistulas more commonly arise from the right coronary artery or left anterior descending artery [17]. Approximately 40% of coronary fistulas drain to the right ventricle, 25%–48% to the right atrium, 17%–38% to the pulmonary artery, 7% to the coronary sinus, and 3%-8% to the left heart [15–20]. In our case, the fistula was arising from the left anterior descending artery and draining into the coronary sinus. Most of the cases remain asymptomatic during infancy and childhood but present later in adulthood due to complications arising from fistulas such as congestive heart failure (due to arterialization of coronary sinus), pulmonary hypertension, angina, or myocardial infarction arising due to coronary steal phenomenon [18]. The increased coronary flow through a fistulous artery adds to the shear stress which causes atherosclerosis. Atherosclerosis can further give rise to aneurysms further complicating the presentation of CAF. IA usually diagnoses CAF; however, multidimensional CCTA is evolving as a reliable noninvasive method of identifying CAF [21]. There is no clear guideline on the management of CAF; some recommend an observation policy for benign fistulas as there are reports of spontaneous closure; however, it is uncommon. Some suggest surgical therapy in order to prevent fistula-related complications [19].

The overall prognosis of CA and absence of IAS is poor as patients who are unable to receive corrective surgery can develop pulmonary hypertension, hypoxia, heart failure, and arrhythmia. The primary objective of surgery is to reconstruct the atrial septum and correct associated congenital abnormalities. Early diagnosis is crucial as the development of Eisenmenger syndrome is a contraindication to surgery [10, 22].

## 4. Conclusion

To the best of our knowledge, this is the first-ever case presentation of a complete absence of the atrial septum diagnosed at 60 years and accompanied with congenital malformations such as coronary artery aneurysm and coronary arteriovenous fistula.

The mainstay therapy is surgery to correct the underlying congenital abnormality; however, due to other associated comorbidities and accompanied malformations, surgery can be challenging. As in our case, it is uncertain whether surgery is of any clear benefit versus conservative measures as the patient did survive till 60 years of age without any corrective measures.

This case also highlights the importance of thorough workup to evaluate for the intracardiac shunt in a patient who has right heart enlargement and development of pulmonary disease in adulthood without a significant history of chronic smoking.
